# Vector control for malaria elimination in Botswana: progress, gaps and opportunities

**DOI:** 10.1186/s12936-020-03375-6

**Published:** 2020-08-26

**Authors:** Tefo Kesaobaka Kgoroebutswe, Ntebaleng Makate, Ulrike Fillinger, Mandla Mpho, Godira Segoea, Peter Onyango Sangoro, Clifford Maina Mutero, Emmanuel Chanda, Davies Ntebela, Mpho Mogopa, Tjantilili Mosweunyane, Theresia Estomih Nkya

**Affiliations:** 1 Department of Biological Sciences, Faculty of Science, University of Botswana, Gaborone, Botswana; 2 International Center of Insect Physiology and Ecology, Nairobi, Kenya; 3 World Health Organization, Botswana Country Office, Gaborone, Botswana; 4 University of Pretoria Institute for Sustainable Malaria Control, Pretoria, South Africa; 5 WHO Regional Office for Africa, Brazzaville, Congo; 6 National Malaria Elimination Programme, Ministry of Health and Wellness, Gaborone, Botswana

**Keywords:** Malaria, Entomology, Integrated vector management, Elimination

## Abstract

Botswana has in the recent past 10 years made tremendous progress in the control of malaria and this informed re-orientation from malaria control to malaria elimination by the year 2020. This progress is attributed to improved case management, and scale-up of key vector control interventions; indoor residual spraying (IRS) and long-lasting insecticidal nets (LLINs). However, insecticide resistance, outdoor biting and resting, and predisposing human behaviour, such as staying outdoors or sleeping outdoors without the use of protective measures, pose a challenge to the realization of the full impact of LLINs and IRS. This, together with the paucity of entomological data, inadequate resources and weak community participation for vector control programme implementation delayed attainment of Botswana’s goal of malaria elimination. Also, the Botswana National Malaria Programme (NMP) experiences the lack of intersectoral collaborations and operational research for evidence-based decision making. This case study focuses on the vector control aspect of malaria elimination by identifying challenges and explores opportunities that could be taken advantage of to benefit the NMP to optimize and augment the current vector control interventions to achieve malaria elimination by the year 2030 as per the Global Technical Strategy for Malaria 2016–2030 targets. The authors emphasize the need for timely and quality entomological surveillance, operational research and integrated vector management.

## Background

Insecticide-based vector control through the use of long-lasting insecticidal nets (LLINs) and indoor residual spraying (IRS) has resulted in a substantial reduction of malaria morbidity and mortality since the year 2000 [1]. In 2017, an estimated 219 million cases of malaria and 435,000 deaths occurred worldwide compared with 239 million cases and 607,000 deaths in 2010 [2]. This reduction in malaria morbidity and mortality has led to many countries, including Botswana, to move from sustained control to elimination as envisaged in the Global Technical Strategy for Malaria 2016–2030 (GTS) targets set by the World Health Organization (WHO) and the Roll Back Malaria partnership (RBMP) [1, 3, 4]. The GTS targets are; (i) to reduce malaria incidence and mortality by at least 90%, (ii) to eliminate malaria from at least 35 endemic countries and (iii) to prevent malaria re-establishment in malaria-free countries by 2030 using vector control as the core intervention [3]. Botswana is among 20 countries identified by the WHO to target malaria elimination by the year 2020, however, the country could not reach this target. In 2019, the WHO reported Botswana to be off track from achieving zero indigenous cases within the year 2020 timeline [5], after the country failed to achieve its 2018 malaria elimination target. The failure to achieve this goal was attributed to weak disease surveillance systems and lack of capacity to optimally implement some of the key interventions, in particular IRS [6]. Moreover, the Southern African region experienced a malaria surge in 2017 attributed to limited entomological surveillance, deficient epidemic detection and rapid response systems and climatic factors such as higher rainfalls and temperatures in the 2016–2017 transmission season [7].

The current malaria control successes and elimination targets are threatened by several factors such as low vector control intervention coverage, weak health systems, and anti-malarial drug and insecticide resistance [1, 8]. Insecticide resistance has been reported globally in malaria-endemic countries with pyrethroid resistance as the most common and widespread in the 77% of 72 countries that have produced insecticide resistance monitoring data [9]. In some countries, major vector populations have been reported to be resistant to all four classes of insecticides (organochlorines, pyrethroids, organophosphates and carbamates) used in public health [9]. In Botswana, insecticide resistance is focal and has been reported for pyrethroids in all the malaria-endemic districts and dichlorodiphenyltrichloroethane (DDT) only in Bobirwa district [6, 9]. In a multi-country study conducted in 5 countries to assess the implication of insecticide resistance to malaria control, evidence from Galabat, Sudan with high LLIN coverage, showed that IRS with an insecticide to which there is resistance provided no additional protection whereas IRS with an insecticide to which there is susceptibility almost halved malaria incidence relative to LLINs alone [10]. Resistance to pyrethroids in Botswana may have an operational impact on the effectiveness of IRS, despite the paucity of data to support such assumptions. Therefore, there is the need to determine the susceptibility status of vectors to insecticides from other classes such as organophosphates and carbamates which may provide Botswana with an opportunity to continue implementation of IRS as the main vector control intervention [11].

In addition to the increasing reports of insecticide resistance, residual malaria transmission (RMT) also poses a major obstacle in achieving the goal of malaria elimination. RMT persists despite the scale-up and effective use of LLINs and IRS due to expression of inherent behaviours, such as biting and resting outdoors which defines the biological limits of these interventions [12–14]. Also, the use of these indoor targeting interventions have led to the altered vector populations, whereby the once-dominant indoor feeding vectors are replaced by outdoor feeding vectors, shifting from intense indoor transmission to residual outdoor transmission [12]. Currently, there are no main interventions that specifically target outdoor biting mosquitoes, even though such interventions will be essential to achieve malaria elimination [15]. Mosquito larval source management (LSM) is the management of water bodies (aquatic habitats) that are potential breeding sites for mosquitoes to prevent the completion of immature development. LSM is a population suppression technique that can be further classified into (i) habitat modification, (ii) habitat manipulation, (iii) biological control, and (iv) larviciding [16]. Larviciding with microbial larvicides has been shown to reduce vector populations in various settings [17–21]. This is achieved by killing mosquito larvae and pupae and/or getting rid of breeding sites, thereby reducing adult density and possibly the number of infective bites per person per year [22]. Larval source management is recommended by the WHO to be used as a supplementary intervention to LLINs and IRS for control of both indoor and outdoor malaria vectors [23].

In response to RMT and insecticide resistance, the WHO and the international community have in recent years increasingly promoted the use of integrated vector management (IVM) as a progressive approach towards sustainable, cost-effective and enhanced malaria vector control [24–27]. The key elements for the successful implementation of IVM are (i) Advocacy, social mobilization, regulatory control for public health and empowerment of communities (ii) Collaboration within the health sector and other sectors through the optimal use of resources, planning, monitoring and decision-making (iii) Integration of non-chemical and chemical vector control methods, and integration with other disease control measures (iv) Evidence-based decision making guided by operational research and entomological and epidemiological surveillance, monitoring and evaluation (iv) Development of adequate human resources, training and career structures at the national and local level to promote capacity building and manage IVM programmes [24]. IVM, though not a new concept, is yet to be adopted by national malaria control/elimination programmes of most countries for control of vector-borne diseases. This has been largely due to a lack of country-specific policies to guide the development and implementation of this approach [25]. Malaria elimination strategies in Botswana combine the use of vector control, case management, epidemic preparedness and response, information, education and communication (IEC) strategies and disease surveillance, monitoring and evaluation [28, 29]. Even though this approach may appear to be an IVM strategy, the country still faces challenges in its implementation to achieve malaria elimination.

This paper describes the path towards malaria elimination in Botswana by identifying challenges that delayed Botswana from reaching the year 2020 malaria elimination target. Furthermore, the paper aims to identify opportunities in vector control aspects that the country could exploit in its efforts towards achieving zero indigenous malaria cases and attain elimination certification by the year 2030.

## Botswana malaria situation analysis

Malaria transmission in Botswana is highly seasonal and unstable, with peak transmission between November and May, during the rainy season, with the highest prevalence recorded in the northern, central and eastern parts of the country [29, 30]. During the years of heavy rainfall, malaria transmission can move southwards causing sporadic malaria cases in the traditionally non-malarious areas [29]. This is because the main malaria vector, *Anopheles arabiensis*, breeds in temporary pools after rains and is highly sensitive to the El Nino Southern Oscillation effects; El Nino (a warm event) and La Nina (a cold event) [31]. As such, inter-annual variation in malaria incidence in Botswana has been attributed to summer rainfalls [32]. *Plasmodium falciparum* is responsible for over 98% of symptomatic malaria cases while *Plasmodium vivax* and *Plasmodium malariae* accounts for the remaining 2% of symptomatic cases in Botswana [28, 29].

Malaria vector control in Botswana started in the mid-1940s, before the launch of the official Botswana National Malaria Control Programme in 1974 [28, 33]. It was based solely on the implementation of IRS with dichlorodiphenyltrichloroethane (DDT) and prevalence was reduced from a high of 73% in Chobe and Ngami Districts in 1944 to 14% in 1974 [33]. Following the ban of DDT use in agriculture [34, 35], which led to a progressive decline in the production of the insecticide, many manufacturing industries closed down. Due to this procurement challenge, the use of DDT for IRS in Botswana was replaced with lambda-cyhalothrin from 1998 until it’s re-introduction in 2010 [6, 11]. Re-introduction of DDT for IRS was attributed to its low cost as well as reports of vector susceptibility to it locally [11, 28, 36] as well as the neighbouring South Africa [37, 38]. Furthermore, this also coincided with reports of emergence of pyrethroid resistance in malaria vectors in the southern Africa region [39–42].

In the year 1992, insecticide-treated nets (ITNs) were piloted in Chobe district and later rolled out to the other malaria-endemic districts (Okavango, Ngami, Boteti, Tutume and Bobirwa) between 1994 and 1998 [11]. The two insecticides (DDT and lambda-cyhalothrin) were used simultaneously from 2010, whereby DDT was sprayed on traditional structures and lambda-cyhalothrin sprayed on modern structures with operational coverage below 77% [6, 11], which is below the minimum recommended 85% per targeted area [43]. Botswana piloted the use of pirimiphos-methyl (an organophosphate) for IRS in Bobirwa district in 2018 and this was extended to all malaria-endemic districts in 2019. IRS, which is implemented in six malaria-endemic districts in Botswana is the main vector control intervention and is supplemented by LLINs (first introduced in 2010 through mass distribution) and larviciding with *Bacillus thuringiensis* serovar *israelensis* [11, 29] (Table 1). Since 2013, larviciding is occasionally implemented in 3 districts whose water bodies are amenable for the intervention [6, 29] and has been shown to reduce larval densities which could reduce malaria risk due to low adult emergence [20, 44].

**Table 1:** Malaria vector control intervention and districts of application [6, 11, 29, 33]

Control intervention	Intervention history	Districts currently applied	Intervention status
IRS	1940–1998: DDT	Okavango, Chobe, Ngami, Boteti, Tutume and Bobirwa	Main intervention
	1998–2009: lambda-cyhalothrin		
	2010–2016: DDT and lambda-cyhalothrin		
	2017: lambda-cyhalothrin and pirimiphos-methyl		
	2018: pirimiphos-methyl		
LLINs	1992: ITNs piloted in Chobe	Okavango, Chobe, Ngami, Boteti, Tutume and Bobirwa	Supplementary intervention
	1994–2009: ITNs roll out in endemic districts		
	2010–2017: LLINs Mass Distribution (every 3 years)		
Larviciding	2010–2012: Piloted in Tutume and Bobirwa	Boteti, Tutume and Bobirwa	Supplementary intervention
	2013–till date: Occasional implementation	Boteti, Tutume and Bobirwa	

Botswana through case management and chemoprophylaxis for pregnant women in malaria-endemic areas and travellers from non-endemic areas reduced malaria case incidence from 280 per 10,000 in 1928 to around 6 per 10,000 in 2010 [45]. The National Malaria Programme (NMP) review conducted in 2009, led to the development of an elimination strategic plan of 2010–2015 given the progress made in malaria control in the country [28]. This plan was formulated to further enhance and sustain efforts in malaria control as the country transitioned to malaria elimination by 2015. However, the mid-term review of the malaria strategic plan 2010–2015 in 2013 informed the extension to 2018 through the extended malaria strategic plan 2014–2018 [29]. This was after the country failed to implement some of the set objectives such as establishment of the insectary and sustainable collaborations with other stakeholders and universal coverage of vector control interventions due to financial inadequacy and limited of advocacy, communication and social mobilization [46]. With guidance from the WHO framework for malaria elimination [47], another malaria programme review was conducted in 2017 [48]. This review recommended the development of an IVM strategy, financial sustainability by seeking funding through innovative mechanisms from other non-health sectors like private companies, capacity building at national and district level and establishment of malaria elimination surveillance, monitoring and evaluation system to enable effective detection and response [48]. These recommendations are part of the objectives to achieve in 2018-2023 national malaria strategic plan [49].

**Fig. 1:**
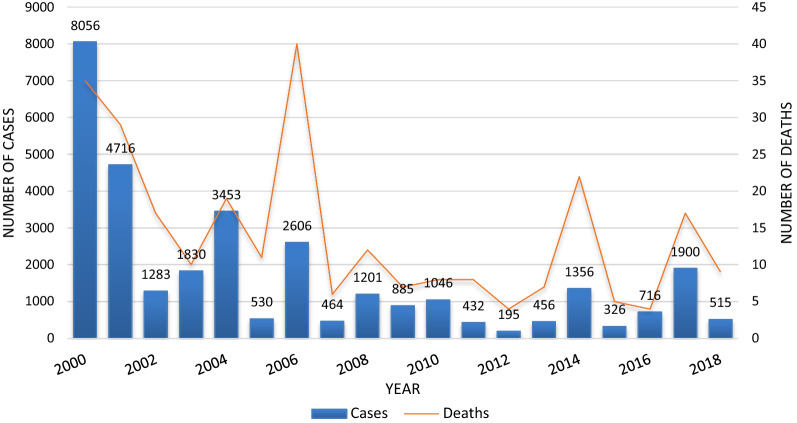
Botswana malaria cases and deaths trends from 2000 to 2018 [6]

Malaria cases and deaths have been declining in Botswana, except for outbreaks in 2006, 2014 and 2017, where there was an upsurge of malaria cases from 530 to 2660 with 40 deaths in 2006, from 456 to 1356 cases with 23 deaths in 2014 and from 716 to 1900 cases with 17 deaths in 2017 (Fig. 1) [6, 29]. Botswana recorded a total of 275 malaria cases and 6 deaths in the 2018/2019 malaria transmission season [50]. For a country to be certified “malaria-free” by the WHO, it must report zero indigenous cases for at least three consecutive years [47]. It is evident from the trends reported here that Botswana does not qualify for malaria elimination certification by the set target of the year 2020, therefore, calling for the reprogramming in line with the GTS targets for elimination by 2030 [3].

## Distribution of dominant and potential vectors in Botswana

**Fig. 2:**
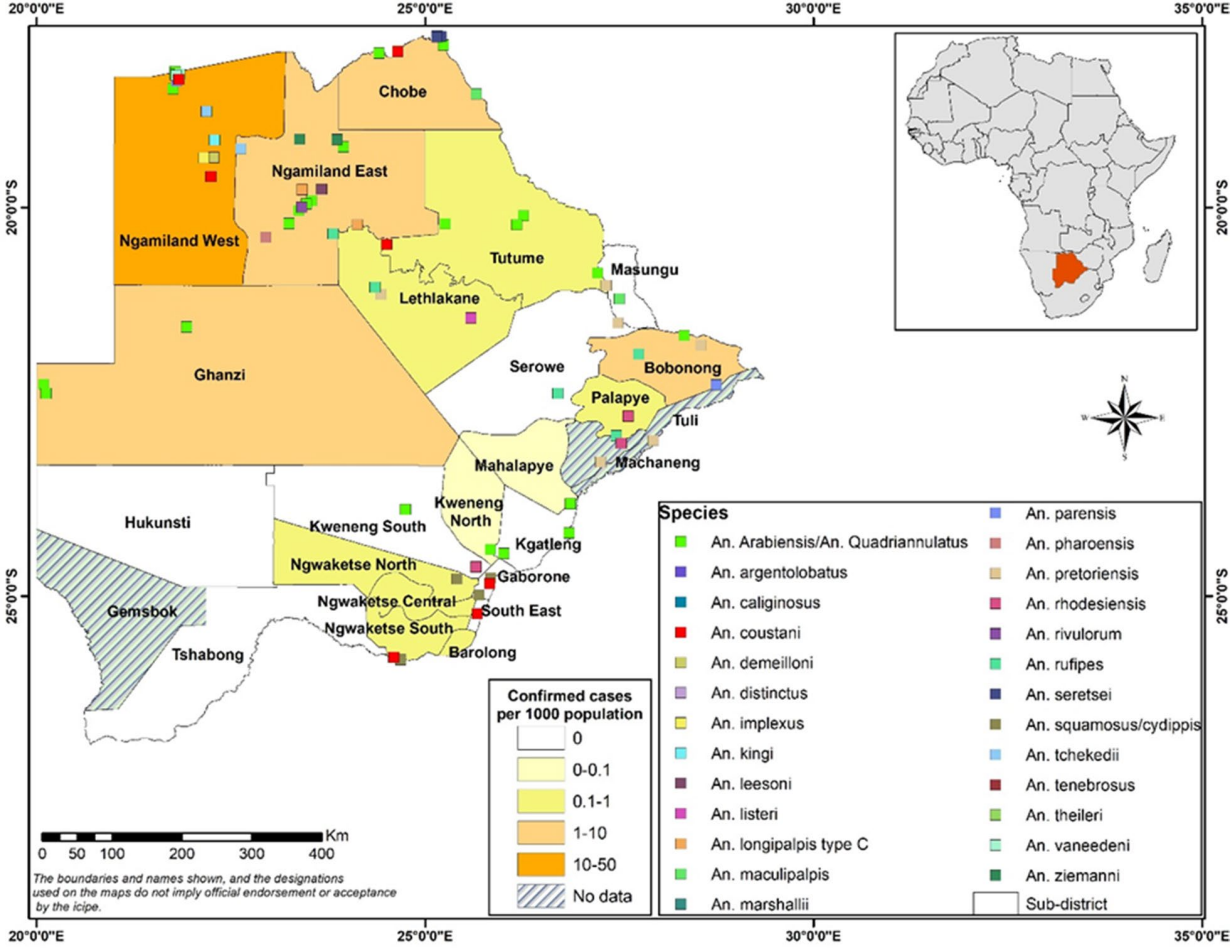
Distribution of Anopheles species and malaria cases per 1000 population in Botswana in 2017 [2, 51–58]

Decades of IRS with DDT decimated the highly anthropophilic and endophilic *Anopheles gambiae* sensu stricto and *Anopheles funestus* leaving *Anopheles arabiensis* as the main malaria vector in Botswana [6, 29]. Even though *An. arabiensis* is widely distributed throughout the country, several other Anopheline mosquitoes have been reported with the earliest reports dating back to 1961 (Fig. 2) [51–58]. However, due to lack of regular entomological surveillance, it is possible that some species may not have been reported while some may no longer be present in the country.

Despite extensive reports that malaria transmission is mostly due to *An. arabiensis* in Botswana, evidence supporting this claim has only been produced in the Okavango region (Ngamiland west) [55]. Moreover, it is not known whether transmission occurs outdoors or indoors [56]. Furthermore, the role of other *Anopheles* species, such as *Anopheles demeilloni, Anopheles marshallii, Anopheles ziemanni, Anopheles longipalpis* type C, *Anopheles parensis* and *Anopheles leesoni* in malaria transmission have not been well documented in Botswana despite reports of these species being infected with *P. falciparum* which implies a possible role in malaria transmission [59–63]. These *Anopheles* species, which play a secondary role in malaria transmission should be recognized for their importance as they may extend and sustain malaria transmission after the primary vectors have been successfully controlled with IRS and LLINs [64]. Secondary malaria vectors are responsible for 5% of malaria transmission in Africa [64]. Therefore, to successfully eliminate and sustain a malaria-free Botswana, it is critical to have adequate information about the ecology and biology of all major and minor malaria vectors in the country.

## *Anopheles arabiensis* and persistence of residual malaria transmission

*Anopheles arabiensis*, the main malaria vector in Botswana [28], displays behaviours that undermine control by intra-domiciliary interventions even when physiologically susceptible. *An. arabiensis* feeding and resting behaviours span from feeding outdoors (exophagy), on animals (zoophagy), resting outdoors (exophilly) to rapidly exiting from houses after entering them when foraging [65–67]. The preference of this species for cattle blood and capability to rest outdoors has been reported in Botswana [55]. Due to feeding and resting behavioural plasticity, it is predicted that malaria will persist longest and will be the most difficult to eliminate from regions inhabited by this species [68]. This is a major challenge for countries in this region in achieving malaria elimination and Botswana is no exception. Therefore, there is a need for innovative tools as well as incorporating already existing population suppressant techniques such as LSM that will target *An. arabiensis* behaviours. A new form of vector control known as attractive toxic sugar bait (ATSB), designed to attract and kill sugar feeding mosquitoes outdoors and have been shown to decrease malaria vector population densities and longevity [69–71] has the potential to supplement IRS and LLINs.

## Malaria vector surveillance

### Insecticide resistance surveillance

Insecticides provide one of the most effective, and best-proven methods of controlling malaria vectors [72] and malaria reduction has mainly been achieved by the use of chemical insecticides through LLINs and IRS [73]. However, the limited number of insecticides approved for public health use and continued over-reliance on pyrethroids for treatment of LLINs and IRS has resulted in selection for insecticide resistance which threatens to derail malaria control [74]. Insecticide resistance is mediated by several mechanisms, mainly; metabolic resistance and target site resistance [75–78].

**Fig. 3:**
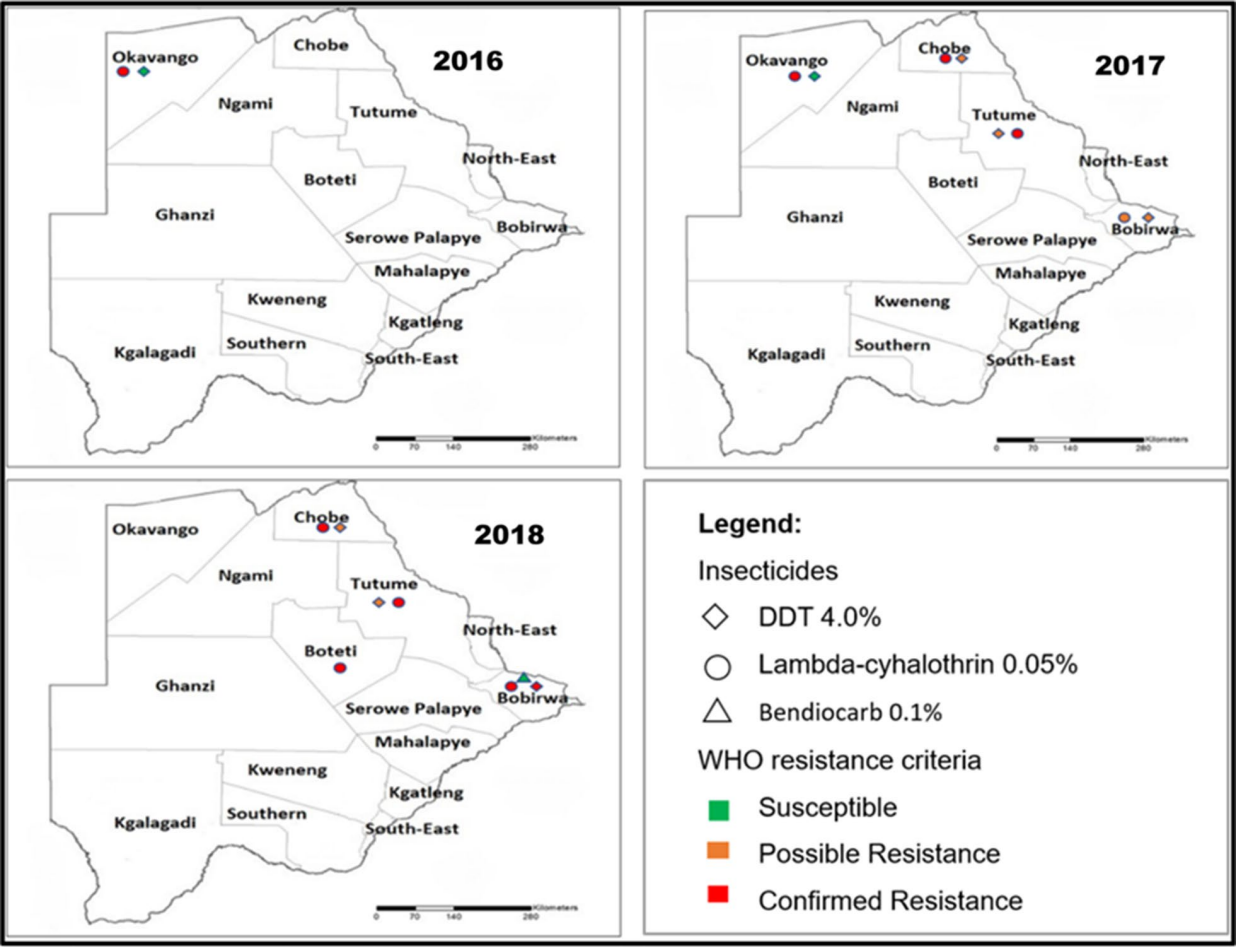
Resistance status in four insecticide classes for *An. arabiensis* from 2016–2018 [6]

Resistance has been reported for lambda-cyhalothrin (pyrethroid), across all the malaria-endemic districts while DDT resistance has only been reported in Bobirwa district in *An. arabiensis* (Fig. 3) [6]. The underlying resistance mechanisms have not been characterized despite the importance of such data in the implementation of the insecticide resistance management strategy. There is need to evaluate the extent of pyrethroid resistance as it has been predicted that malaria incidence may increase as a result of lower overall community protection over the lifetime of the pyrethroid treated net as a result of insecticide resistance [79].

Botswana has recently formulated an insecticide resistant management strategy (IRMS) to use appropriate strategies to manage resistance, achieve efficient vector control and attain malaria elimination [6]. This strategy presents an opportunity for the country to preserve the effectiveness of all currently used insecticides and manage the development of insecticide resistance given that only DDT and pyrethroids have been used for IRS in Botswana [6]. The strategy presents a total of four specific objectives; (i) strengthening the capacity for timely generation, interpretation and use of entomological data for vector control decision-making process, (ii) establishing and sustaining collaboration within the health sector and with other sectors and other stakeholders in the prevention and management of insecticide resistance (iii) strengthening operational research on investigating the spread and mechanisms of insecticide resistance and (iv) implementation of effective insecticide resistance management approaches tailored for Botswana. The implementation of this strategy will require more resources and as such more commitment of funds from the government as well as other national and international partners, such as WHO, RMBP, Clinton Health Access Initiative (CHAI), Global Fund and Southern African Development Community (SADC).

### Entomological surveillance

**Fig. 4:**
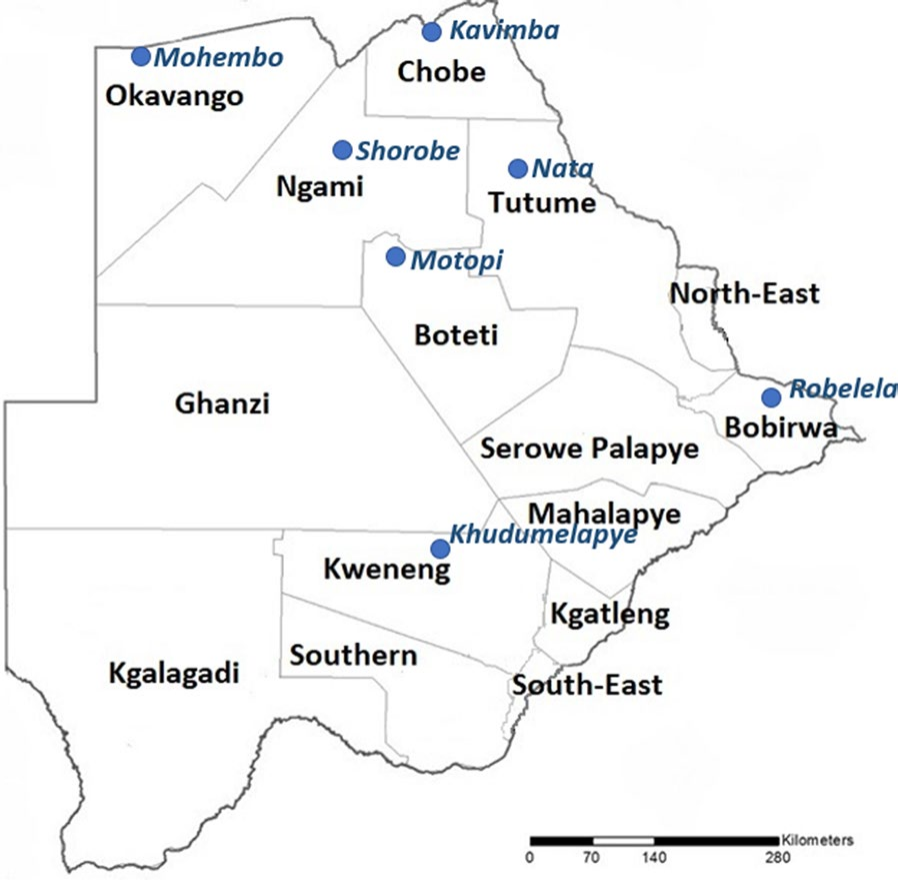
Malaria entomological surveillance sentinel sites in Botswana [6]

One of the pillars of the GTS for malaria recommends adequate entomological surveillance and monitoring which include a periodic assessment of vector species present in a given region, their abundance and seasonality, time and place of biting, resting and host preference, insecticide susceptibility status and underlying resistance mechanisms to predict vulnerability to interventions and maximize the impact of vector control [3]. Following the 2009 malaria programme review, Botswana set up seven sentinel sites in 2009, to carry out entomological surveillance to generate data which is essential in predicting the efficacy of interventions against vectors (Fig. 4) [6].

These sentinel sites are not fully functional due to lack of capacity in both human resources and infrastructure, hence, Botswana still lacks critical data on local vector species and their susceptibility to insecticides [56]. Moreover, there are no clear guidelines for mosquito sampling and rearing, analysis, interpretation and use of entomological data [6, 29]. The GTS target to prevent malaria re-establishment in malaria-free countries by 2030 using vector control as the core intervention [3]. To achieve this target, it is crucial to have up to date malaria vector data, which for Botswana this is lacking. To fill this knowledge gap, collaborations between the NMP, research institutions and universities is urgently needed if elimination is to be achieved and sustained.

Vector ecology has been identified as essential for malaria elimination as numerous ecologically imposed obstacles allow vector populations to resist or evade interventions thus limiting their effectiveness [80, 81]. National Malaria Programme Review of 2009 [28], among others, recommended the scale-up of entomological surveillance as the programme was re-orienting from malaria control to elimination in Botswana, the same was echoed in 2017 by Tawe et al. [56]. Sri Lanka, a country that received its malaria elimination certificate in 2016, among the various strategies, implemented rigorous entomological surveillance in over 50 sentinel sites and spot surveillance checks during changes in environmental conditions favour vector breeding and anthropogenic activities [82]. This enabled implementation of a highly targeted and situation-specific vector control programme coupled with larval source management played an important role in malaria elimination in Sri Lanka [82]. Furthermore, Algeria received its elimination certification in 2019, among various strategies, by implementing geographic information system mapping and entomological surveillance to document the movement of mosquito vectors carrying malaria in the southern region and border areas [83]. Botswana could learn from these countries and adapt their strategies for routine surveillance to enable implementation of highly targeted and situation-specific vector control interventions and cross-border entomological surveillance.

## Resources for malaria vector control

### Human and infrastructural resources

Botswana also faces a challenge of inadequate human and infrastructural resources for malaria control at all levels. The Botswana NMP is comprised of 1 Entomologist, 3 Technicians and 5 field assistants which are not adequate for strengthening entomological surveillance in the whole country [6]. The country also lacks functional infrastructures to carry out standard entomological evaluations, such as lack of insectary and a specialized laboratory for molecular analysis [6]. Years of successful malaria control was derailed by lack of personnel to carry out good coverage and surveillance activities which led to epidemics in southern Turkey in 1977 and 1993–1996 [84]. The resurgence of malaria in Mauritius in the 1970s and outbreaks in 1998–99 in Turkmenistan were attributed to lack of human and financial resources.

In Sri Lanka, which received malaria elimination certification in 2016, malaria control activities were decentralized to regional malaria officers with each region comprising of Public Health Inspectors, Public Health Laboratory Technicians, Public Health Field Officers, and an entomology team for vector surveillance and vector control [82]. Botswana’s National Plan for Insecticide Resistance Prevention and Management in Malaria Vectors 2018–2021 aims to strengthen local capacity at the districts level and in sentinel sites to support entomological surveillance and insecticide resistance monitoring after conducting a comprehensive vector control needs assessment in line with the WHO framework [6]. The country also aims to establish a functional insectary at the entomology unit in Francistown and conduct insecticide resistance monitoring at least once in a year in all sentinel sites [6]. Two years into this 4-year strategic plan, no progress was achieved in this regard and this partly contributed to the failure to achieve elimination by 2020 and maybe so for the next few years to come.

Botswana NMP also aims to collaborate with local and international universities and research institutions for capacity building [49]. This collaboration will allow access to well-established infrastructures such as laboratories and insectaries to coordinate efforts for the successful elimination of malaria. Botswana should use the available training resources such as training modules developed by the WHO to improve the capacity of personnel in essential aspects of malaria entomology and vector control [85, 86]. Not only that, the country should also commit funding for building human resource capacities and foster collaborations with established African IVM institutions to heighten and sustain in-country implementation of IVM [27]. Sustained capacity building and strong supervision and mentoring are key to successful elimination. This requires a robust training and staff retention plan to ensure and sustain the quality deployment of interventions [87].

### Intersectoral collaboration

IVM seeks to improve the efficacy, cost-effectiveness, ecological soundness and sustainability of vector control. This guided by operational research and subject to routine monitoring and evaluation through the collaboration of the health sector with various stakeholders’ (such as public and private agencies and community) [25]. An IVM-based approach, however, demands more effective planning and decision-making at the lowest possible administrative level, and it should not only be cost-effective with indicators of impact monitoring on vector populations and pathogen transmission but should also be sustainable and compatible with local health systems [24]. Intersectoral collaboration has been recommended as one of the key elements of IVM after the recognition that effective vector-borne disease control is not the sole responsibility of the health sector [24]. There is lack of strong and efficient collaboration between the Ministry of Health and Wellness malaria programme and other relevant government sectors/ministries, the private sector, the community/public and academia who are all essential in malaria control and elimination in Botswana [29]. Several studies suggest there is a positive impact of intersectoral collaboration in malaria control and elimination [21, 88–93]. Effective inter-sectoral collaboration is, however, influenced by factors such the approach, resources, relationships, management and shared vision [94].

In the past decade, a drop in malaria cases and mortality in Botswana was observed, however, these declines have been stalled due to weak disease surveillance systems and lack of capacity to optimally implement some of the key interventions [6, 11]. IVM is relevant in strengthening vector control especially in areas where malaria control has been successful but currently stalled and further efforts are required to go from sustained low transmission situations to malaria elimination [25]. However, there are challenges in the implementation of IVM such as limited technical and infrastructural capacity, untimely and improperly conducted entomological surveillance and insecticide resistance monitoring, limited channels for effective involvement of communities and other sectors and change from vertical management to a multi-sectoral approach [27]. Botswana can benefit from an IVM-based approach in malaria control, but the country would need to go beyond formulating strategies and invest in research that will inform current challenges and guide the deployment of IVM. It is essential that the country also establishes a malaria vector control advisory committee or a malaria vector technical working group with membership drawn from key sectors to ensure successful and effective collaboration, assess progress and make decisions in line with emerging evidence from operational research and all relevant activities related to malaria vector control [29].

Malaria transmission is interconnected across borders and is, therefore, dependent on cross border collaboration and engagement. Botswana is a landlocked country that has to recognize that the progress or failure of one country’s efforts to eliminate malaria is connected to the success of other countries in the region. For Algeria, to achieve its malaria elimination certification in 2019, the country strengthened cross-border collaborations with its neighbours Mauritania, Tunisia, Niger, Mali, and Libya [83]. Beyond country intersectoral collaboration, Botswana is a member of Elimination 8 (E8) countries, a regional initiative established in 2009 by the Southern African Development Community (SADC) to coordinate a collaborative effort, led by the Ministers of Health in eight countries; Botswana, Namibia, South Africa, Eswatini, Angola, Mozambique, Zambia, and Zimbabwe to jointly plan and execute a regional malaria elimination strategy and mitigate cross border transmission which presents the threat of re-establishment of infection in areas aiming to interrupt transmission [95]. However, the initiative is currently faced with several challenges of its own, such as lack of standardized regional routine surveillance systems to determine the ecology and distribution of the vector species present and poor vector resistance monitoring and documentation [96].

Botswana has made tremendous success in malaria control, despite limited research in malaria [6, 28]. Research to develop new or improved anti-malaria interventions (drugs, vector control tools, diagnostics and vaccines), to inform policy decisions on the type of interventions and programs best suited to the local context and to understand the use and the effectiveness of interventions in the field is essential for effective malaria control and elimination [97]. Therefore, the country could benefit from investment in research and integration of suitable intervention(s) based on evidence from the findings of the research on outdoor control interventions of *An. arabiensis* and other possible secondary vectors. Besides LSM, deployment of other outdoor control interventions such as ATSBs, space spraying, oviposition deterrents, zooprophylaxis and ivermectin human administration using an IVM-based approach to supplement IRS and LLINs has been identified as essential to successfully control and eliminate malaria [15, 84, 98]. Malaria elimination is a long and strenuous road which requires sustainable and integrated approaches and interventions, the use of new tools for malaria control and elimination, as well as building and sustaining human resource capacity, surveillance and health systems, are crucial for achievement [84].

## Conclusion

This article highlights the gaps and challenges that need to be addressed to propel Botswana towards malaria-free status by 2030. Botswana’s malaria elimination efforts face challenges and there are gaps, which if addressed sooner than later, could result in achieving malaria elimination by the year 2030. Botswana has to go beyond identifying challenges in malaria strategic plans and actually implement and even benchmark from countries that have managed to eliminate malaria. The fact that there are malaria cases still recorded in hundreds and deaths in units means that Botswana needs to evaluate its approach towards the disease and implement more effective IVM strategies.

Botswana needs to build local human resource capacities in the different fields of public health including those relevant to malaria such as medical entomology, epidemiology, information technology and social science by either training or employing the already trained personnel. It is only through adequate human resources, that the Botswana NMP could enhance the efforts towards malaria elimination. Botswana needs to also extensively monitor and evaluate its vector control programme to ensure the sustainability of the interventions. The paper focused mainly on vector control even though sustainable vector control is highly dependent on other aspects such as financial adequacy and community participation. Therefore, communities should be engaged to identify reasons for low interventions uptake and how best to work together towards malaria elimination.

There is also a need to establish strong and sustainable collaboration with research institutions and academia such as the University of Botswana and Botswana International University of Science and Technology which have established human and infrastructural capacity that includes well-equipped laboratories for molecular analysis as well as entomological evaluations such as susceptibility tests for insecticide resistance monitoring. The NMP should fast track collaborations with research institutions to help acquire funds through innovative research ideas from funding agencies thereby facilitating evidence-based malaria vector control. The NMP should assess the impacts of incorporating outdoor vector control interventions such as ATSBs as several trials of ATSBs as an outdoor mosquito control tool have been very successful. Also, the international collaboration with neighbouring member states, academic institutions and organizations, will be of added benefit to Botswana in the road towards elimination. Malaria elimination has to be made a priority at all levels with strong political support more so given that current strategies and approaches have created communities fatigue towards interventions and this thwarts the efforts towards elimination.

## Data Availability

Not applicable.

## Abbreviations

ATSB: Attractive toxic sugar bait
DDT: Dichlorodiphenyltrichloroethane
GTS: Global Technical Strategy for Malaria 2016–2030
IRS: Indoor residual spraying
IVM: Integrated vector management
LLINs: Long-lasting insecticidal nets
NMP: National Malaria Programme
RMT: Residual malaria transmission
WHO: World Health Organization
